# Dynamic behavior of a nematic liquid crystal with added carbon nanotubes in an electric field

**DOI:** 10.3762/bjnano.9.25

**Published:** 2018-01-22

**Authors:** Emil Petrescu, Cristina Cirtoaje

**Affiliations:** 1 University Politehnica of Bucharest, Department of Physics, Splaiul Independenţei 313, 060042, Bucharest, Romania

**Keywords:** Fréedericksz transition, nanotubes

## Abstract

The dynamic behavior of a nematic liquid crystal with added carbon nanotubes (CNTs) in an electric field was analyzed. A theoretical model based on elastic continuum theory was developed and the relaxation times of nematic liquid crystals with CNTs were evaluated. Experiments made with single-walled carbon nanotubes dispersed in nematic 4-cyano-4’-pentylbiphenyl (5CB) indicated a significant difference of the relaxation time when compared to pure liquid crystal. We also noticed that the relaxation time when the field is switched off depends on how long the field was applied. It is shorter when the field is switched off immediately after application and longer when the field was applied for at least one hour.

## Introduction

The increased interest for nanomaterials in different domains such as chemistry, medicine or engineering, makes their characterization quite necessary. This might be the reason why there are so many research papers presenting new materials and new experiments regarding their behavior in different external electric, magnetic or laser fields [1–8]. In many cases, the chemical or physical properties of microparticles or nanoparticles are different from those of the corresponding bulk materials. Hence, new measurement methods and theoretical models have to be developed for their characterization [5–6,9–11].

When nanoparticles are inserted in liquid crystals, nematic molecules are attached to the particle surface due to anchoring forces. Experimental studies revealed that carbon nanotubes have a strong interaction with liquid crystal molecules and align themselves parallel with the long axis of the nematic [12–14]. Under the action of an external magnetic or electric field above a critical threshold, nematic molecules collectively change their orientation. This is called the Fréedericksz transition [15–16] and the molecular movement is characterized by the relaxation time. Experimentally it can be determined by measuring the intensity of a light beam traversing the sample as a function of the time. A theoretical model based on elastic continuum theory, in which the interaction between carbon nanotubes and LC molecules is similar to the one described by Burylov et al. [17], was used to calculate the relaxation times when the field is switched on or off, and the values were in good agreement with experimental data. The experiments were performed on a 0.1% volumetric fraction of single-walled carbon nanotubes (SWCNTs) in 4-cyano-4’-pentylbiphenyl (5CB) liquid crystal (LC).

## Theoretical Background

**Figure 1:**
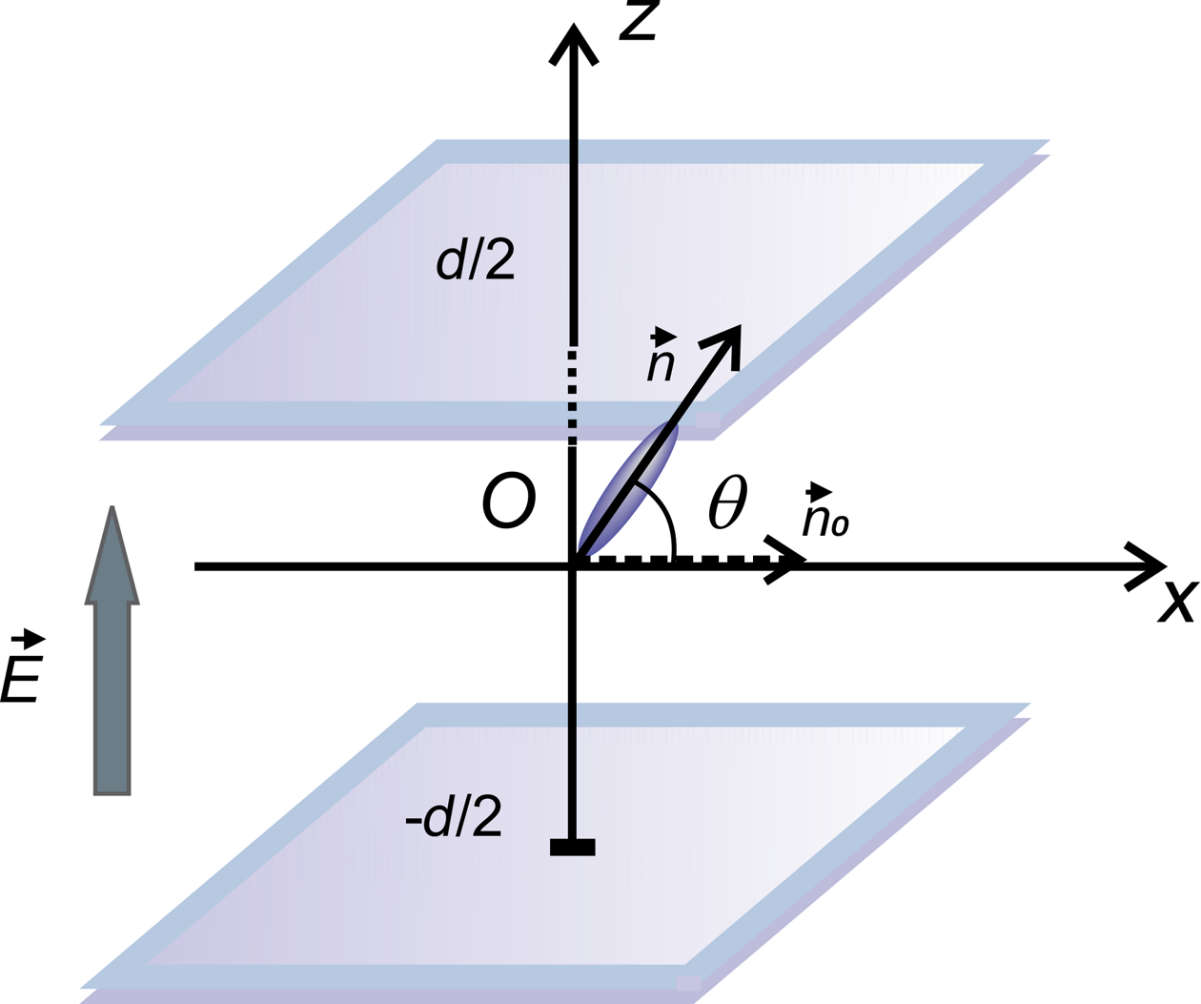
Liquid crystal molecular orientation inside a LC cell exposed to an electric field. 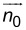 is the undisturbed molecular director and 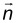 is the molecular director when the electric field is applied.

When a liquid crystal with positive dielectric anisotropy is exposed to an external electric field higher than the critical Fréedericksz transition threshold, its molecules have a tendency to orient their director parallel to the applied field, deviating by an angle θ from their initial direction (Figure 1).

This deviation reaches its maximum value (θ_m_) after a time period called the relaxation time. Consequently, the refractive index of the cell is also changing:

[1]
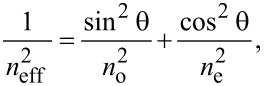


where *n*_o_ and *n*_e_ are the ordinary and extraordinary refractive indexes. Thus, the path difference between the ordinary and extraordinary rays for a cell with thickness *d* can be written as:

[2]
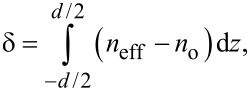


with the corresponding phase difference Δφ = (2πδ)/λ. For a planar aligned cell in which small values of θ are considered, this phase difference becomes:

[3]
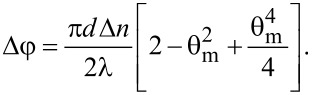


The intensity of the beam traversing through the sample placed between two cross polarizers is:

[4]
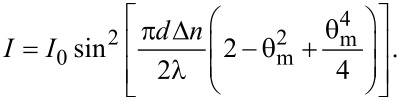


The time dependency of the emergent beam intensity is given by the maximum deviation angle (θ_m_). For the analysis of this angle we must take into account all the interactions of the nematic molecules with the surrounding molecules, with the carbon nanotube surfaces, with the glass support and with the applied field. By applying the elastic continuum theory we can write the free energy of a system consisting of a liquid crystal and SWCNTs in electric field as:

[5]
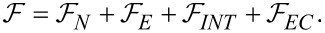


**Figure 2:**
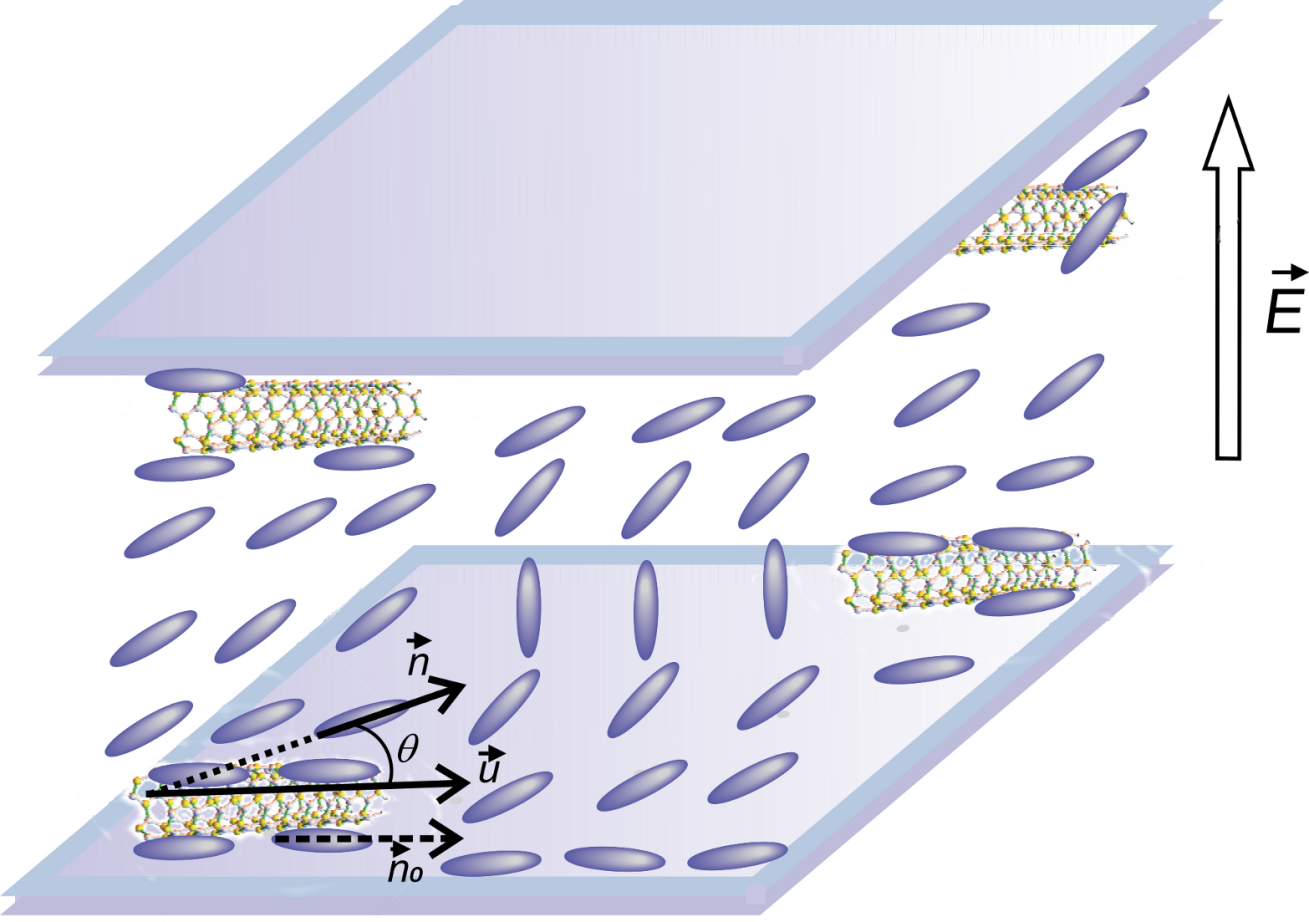
Carbon nanotubes in a liquid crystal cell exposed to an electric field.

Here 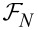 is the elastic free energy of liquid crystal, 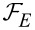 is the interaction free energy of liquid crystals with the applied electric field, 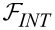 is the interaction free energy between LC and SWCNTs and 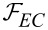 is the interaction free energy of the carbon nanotubes with the applied field. When a planar aligned cell is considered and the electric field is perpendicular to the support (i.e., parallel to the *z*-axis), the molecular orientation is characterized by the distortion angle θ between the director and the *x*-axis (Figure 1). Thus, the free energy of the liquid crystal is:

[6]
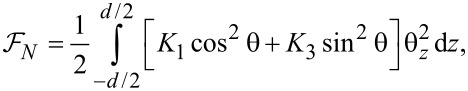


where θ*_z_* = ∂θ/∂*z*, *d* is the cell thickness and *K*_1_, *K*_3_ are elastic constants. The interaction free energy of a liquid crystal with the electric field (Figure 2) is:

[7]
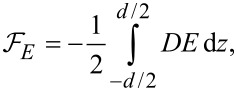


where

[8]
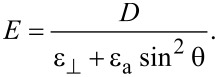


Here 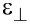 is the transverse component of the dielectric permittivity and 
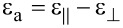
 is the dielectric anisotropy of the liquid crystal.

Since *D*, the electric displacement, is constant, the voltage between the electrodes can be written as:

[9]
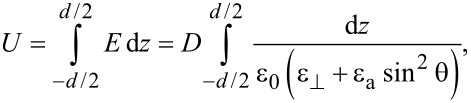


and Equation 7 becomes:

[10]
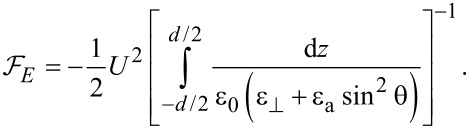


**Figure 3:**
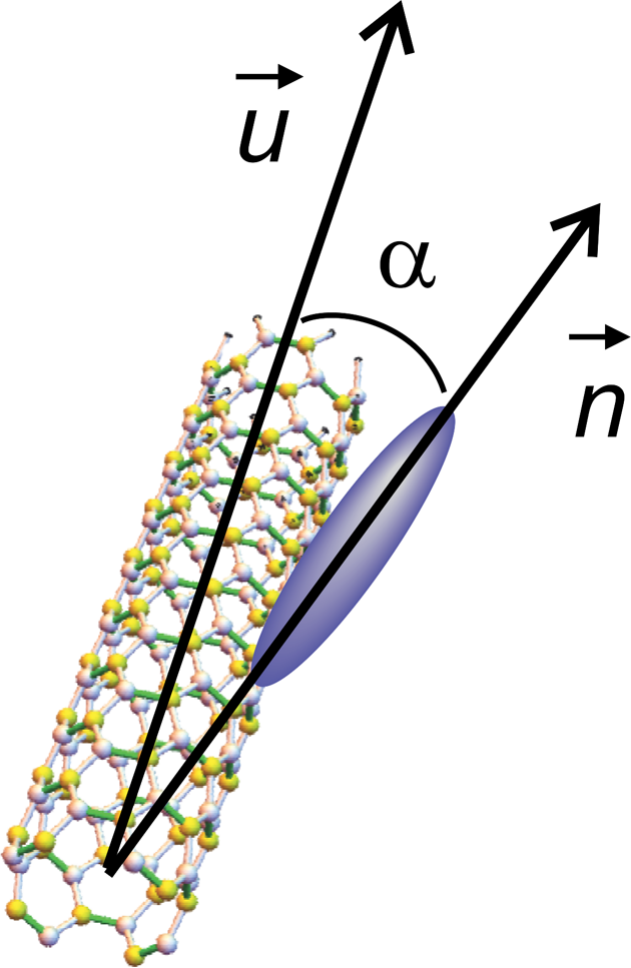
Orientation of a liquid crystal molecule on a CNT surface.

If we consider the interaction free energy between nanoparticles and nematic liquid crystal molecules given by the model proposed by Burylov and Zakhlevnykh [17], we get:

[11]
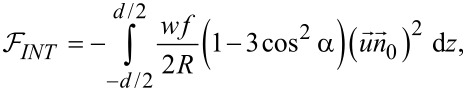


where *f* is the volumetric fraction of nanotubes, *R* is the nanotubes radius, *w* is the average anchoring energy density at the nematic–nanotubes interaction surface, 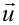 is the unit vector of the CNT axis, α is the angle between the surface of nanotubes and the NLC molecules (Figure 3), 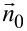 is the direction of the nematic molecular director outside the nanotubes range of action.

Previous experiments developed by Lynch and Patrick [12], and by Dierking and co-workers [13–14] proved an alignment of CNTs parallel to the liquid crystal molecules, so we may assume that the anchoring angle α is neglectable. Thus, Equation 11 becomes:

[12]
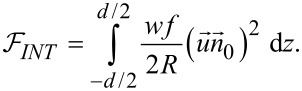


**Figure 4:**
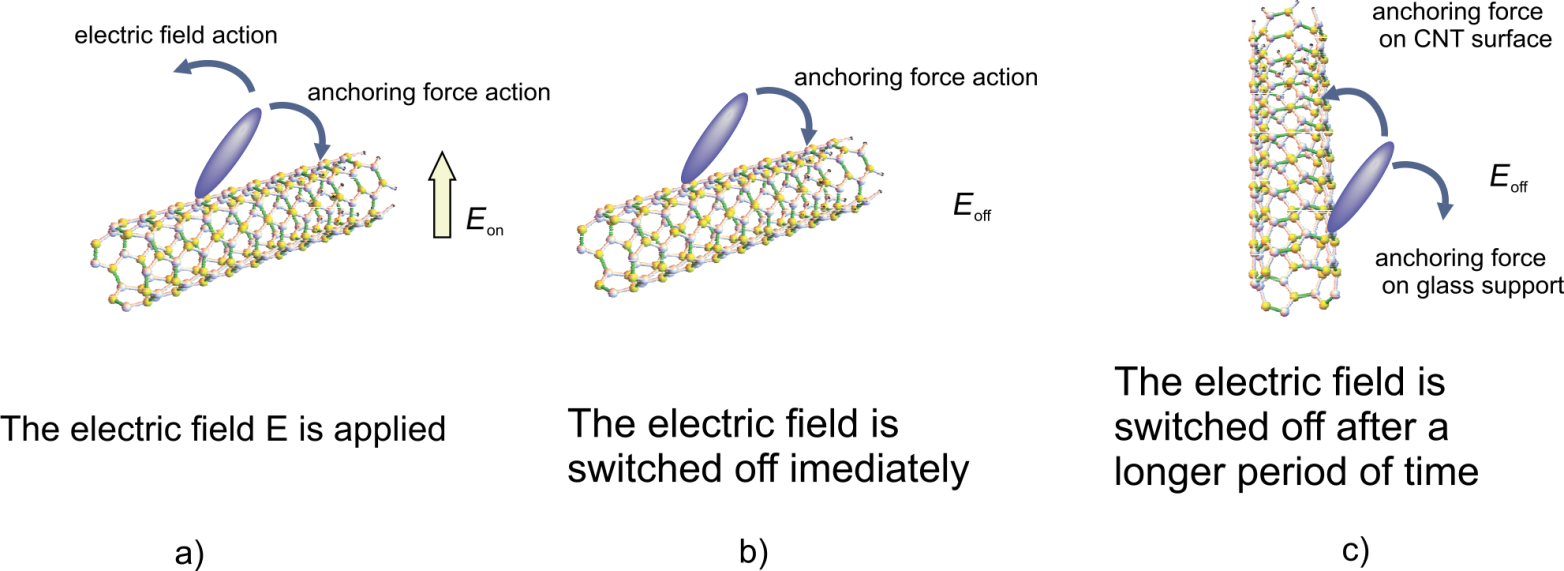
Relative orientation of a liquid crystal molecule on a carbon nanotube surface. a) The electric field is applied, b) the electric field is switched off immediately, c) the electric field is switched off after a longer period of time.

When exposed to an external electric field higher than the critical Fréedericksz transition threshold the molecules orient themselves parallel to the field. Considering their large size (up to 2 μm length) and low concentration (0.1% volumetric fraction) we assumed that the carbon nanotubes will not change their orientation as fast as the liquid crystal molecules. The case of strong anchoring of nematic molecules to the nanotubes surface and a homogeneous alignment of the cell was considered. Thus, in the absence of any external fields, the nematic director and carbon nanotubes axis are parallel to the *x*-axis. When the electric field is applied on a LC + SWCNT cell, the molecules change their orientation tending to become parallel to the field, but the carbon nanotubes remain still parallel to the cell support (Figure 4a). If the field is switched off immediately, the nematic molecules return to their original position, “helped” by the anchoring forces on the nanotube surface, leading to a shorter response time compared to the liquid crystal sample (Figure 4b). If the voltage is applied for a long period of time (at least 1 hour) the nanotubes align parallel to the field, due to their positive anisotropy [18–21]. When the field is then switched off, the LC molecules return to their original position, but their movement is slowed by the same anchoring forces acting on the nanotube surfaces (Figure 4c). In this case, a longer relaxation time is obtained.

When reaching the threshold value, θ increases by a small amount. Consequently, we consider only small values of θ to evaluate the free energy:

[13]
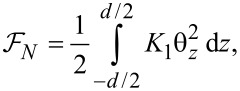


[14]
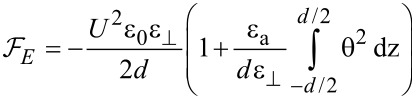


and

[15]
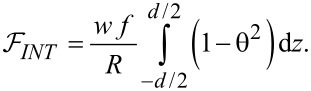


Using Equations Equation 13–Equation 15 the total free energy becomes:

[16]
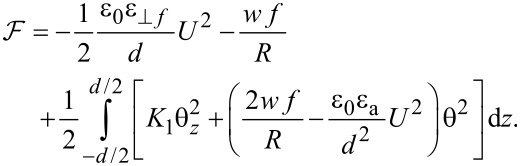


As the first two terms in Equation 16 are constant we may neglect them and introduce a new free energy density term:

[17]
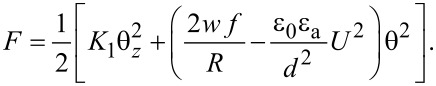


Using the Euler–Lagrange equation:

[18]
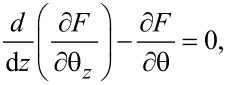


and considering the boundary conditions for a strong anchoring on the cell support, θ(−*d*/2) = θ(*d*/2) = 0, we obtain the threshold voltage for the Fréedericksz transition:

[19]
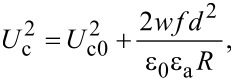


where

[20]
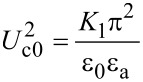


is the Fréedericksz transition voltage of the nematic. Therefore, when the electric field is applied for a short period of time, the critical voltage for Fréedericksz transition increases in the cell containing nanotubes (Equation 19) when compared to those containing the pure nematic (Equation 20).

For the theoretical study of the dynamic evolution of the LC + SWCNT system we considered a constant electric field inside the cell, an approximation that works very well for weak fields. First, we consider the moment in which the electric field is switched on. When dynamic evolution is analyzed, we must also take into account the liquid crystal rotational viscosity which slows the molecular reorientation. Thus, an additional dissipative term must be added:

[21]
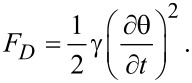


The free energy density of the nematic and CNT cell now becomes:

[22]
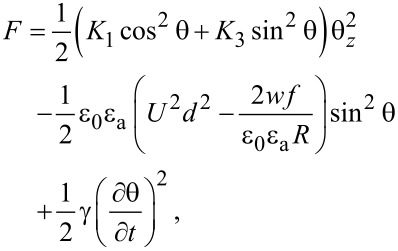


and the corresponding Euler–Lagrange equation is:

[23]
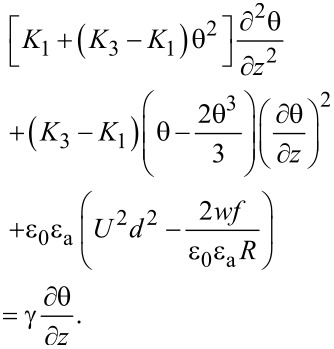


Assuming the deviation angle inside the cell as

[24]
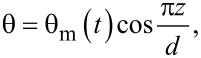


where θ_m_ is the deviation angle in the middle of the cell. Its time evolution is:

[25]
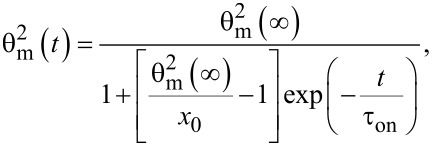


where θ_m_(∞) is the maximum value of the deviation angle when the electric field is applied for a very long period of time, *x*_0_ is a constant parameter and τ_on_ is the relaxation time when the field is switched on. Following a similar procedure as in [22–24], we get:

[26]
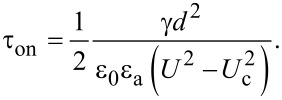


We consider two different cases for switching the field off: First, when the field is switched off immediately, the nanotubes are still aligned along the *x*-axis. In this case the relaxation time will be smaller than that of a pure nematic because the interaction forces between nematic molecules and carbon nanotubes pull the molecules back to a planar alignment. The free energy density results from Equation 22 in which the applied voltage is *U* = 0:

[27]
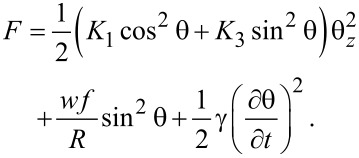


The time variation of the maximum deviation angle is:

[28]
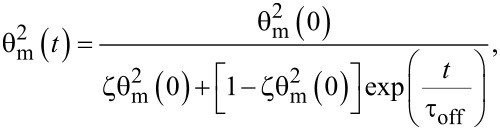


where 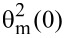 is the initial value of the maximum deviation angle, ζ is a parameter depending on the carbon nanotube concentration, the interaction energy and the physical properties of nanotubes and liquid crystal. The relaxation time when the field is switched off immediately after its application to the SWCNT containing sample is:

[29]
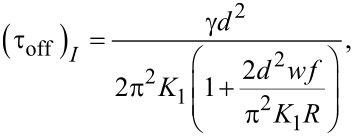


while for the pure liquid crystal it is:

[30]
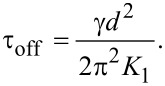


It can be seen the relaxation time for LC + SWCNTs is shorter than that of the pure nematic. The second case we consider is the application of the field for a period of time that is long enough to allow the carbon nanotubes to align themselves in parallel to the field. In this case the free energy density is:

[31]
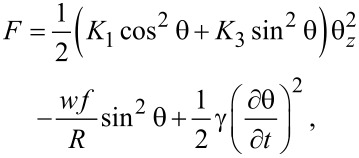


and the relaxation time is:

[32]
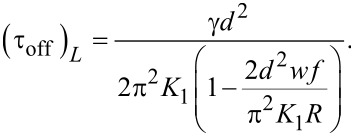


As shown in Equation 32 it is longer than that of the pure nematic.

## Experimental

**Figure 5:**
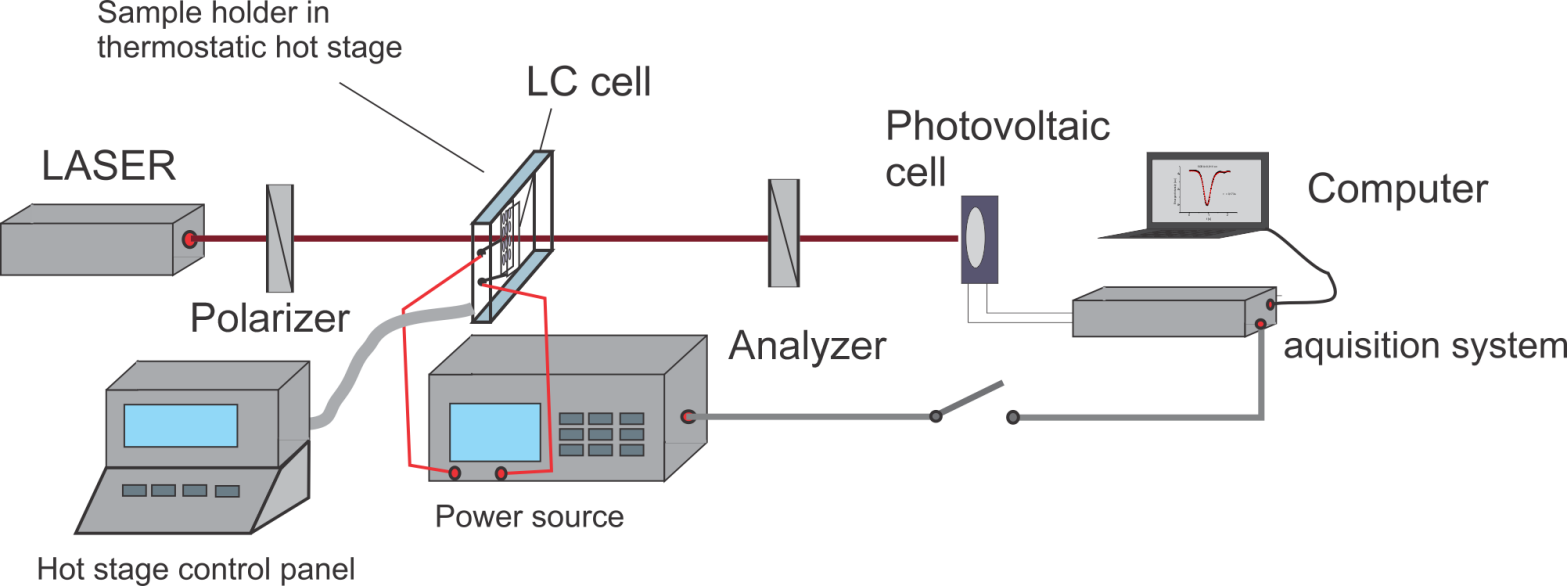
Experimental setup for the dynamic study of LC + SWCNTs in an electric field.

We used SWCNTs provided by Aldrich having a diameter of 1.2–1.5 nm and a length of 2 up to 5 μm. A 0.1% volumetric fraction of nanotubes was mixed with 5CB and sonicated for several hours at 40 °C. The mixture in the isotoropic phase was used to fill a planar aligned cell with a thickness of 15 μm from Insteck also warmed at 40 °C. Then the samples were slowly cooled down to 29 °C in a holder placed inside a Mettler Toledo stage. The terminals of the holder were connected to a HIOKI RLC power source from which a 10 kHz ac voltage was applied. A 632.8 He–Ne laser sent a beam through the sample placed between two crossed polarizers (Figure 5). A Thor Lab photovoltaic cell was used to record the emergent beam through the sample.

## Results and Discussion

**Figure 6:**
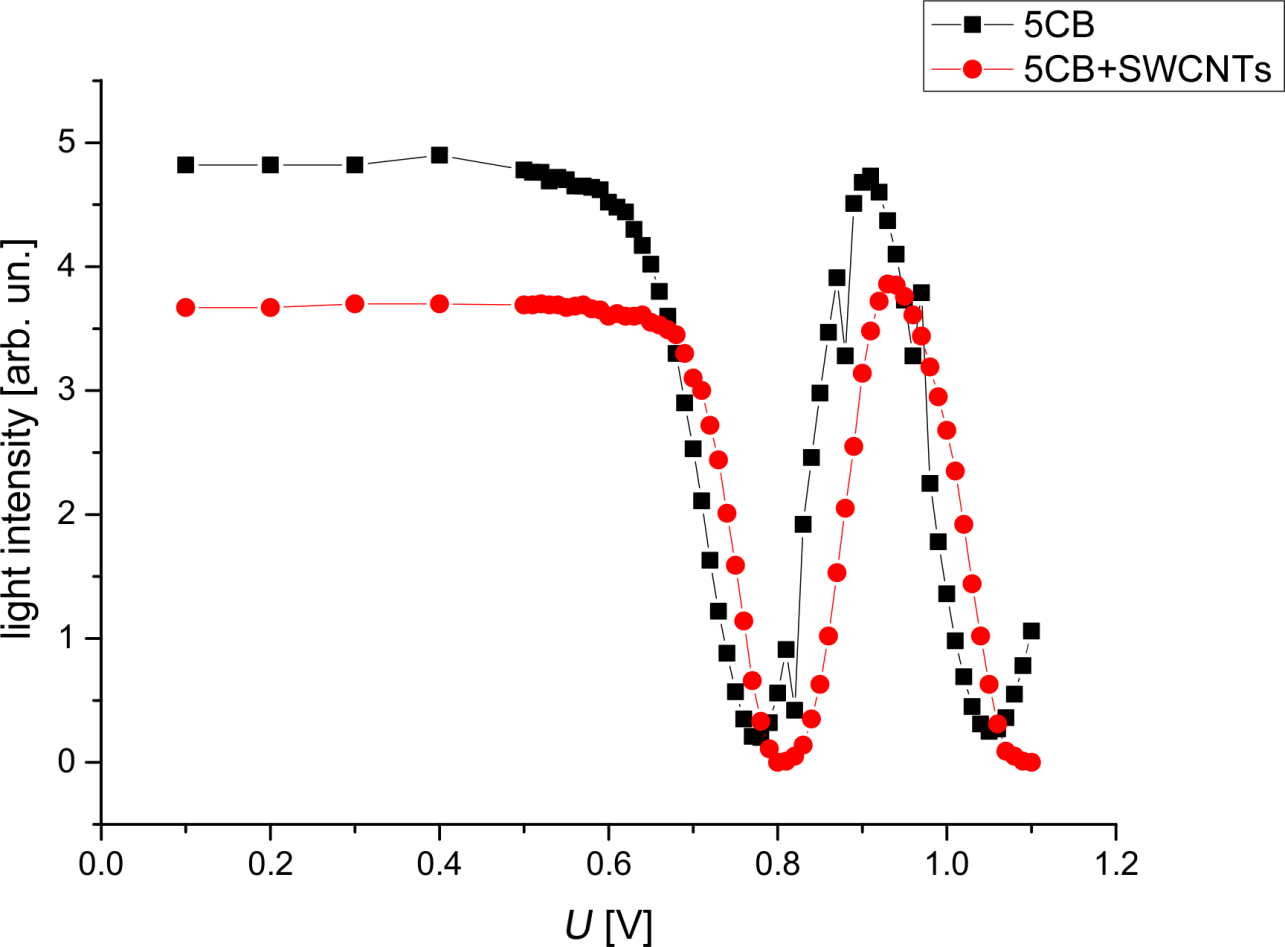
Fréedericksz transitions for 5CB and 5CB + SWNCTs.

By slowly increasing the electric field applied, the Fréedericksz transition was determined both for LC and LC + SWCNTs (Figure 6).

**Figure 7:**
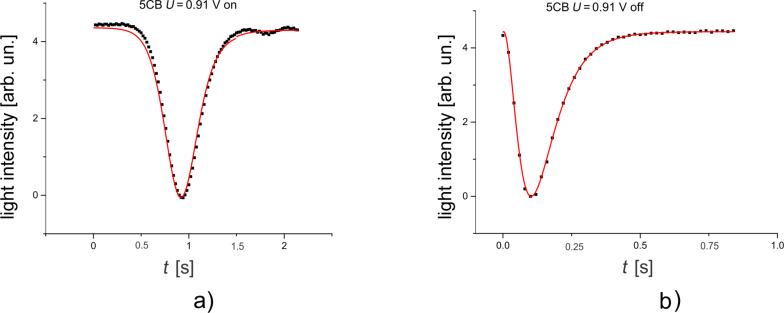
Variation of the intensity of a light beam traversing a cell containing pure 5CB. a) The field is switched on, b) the field is switched off.

**Figure 8:**
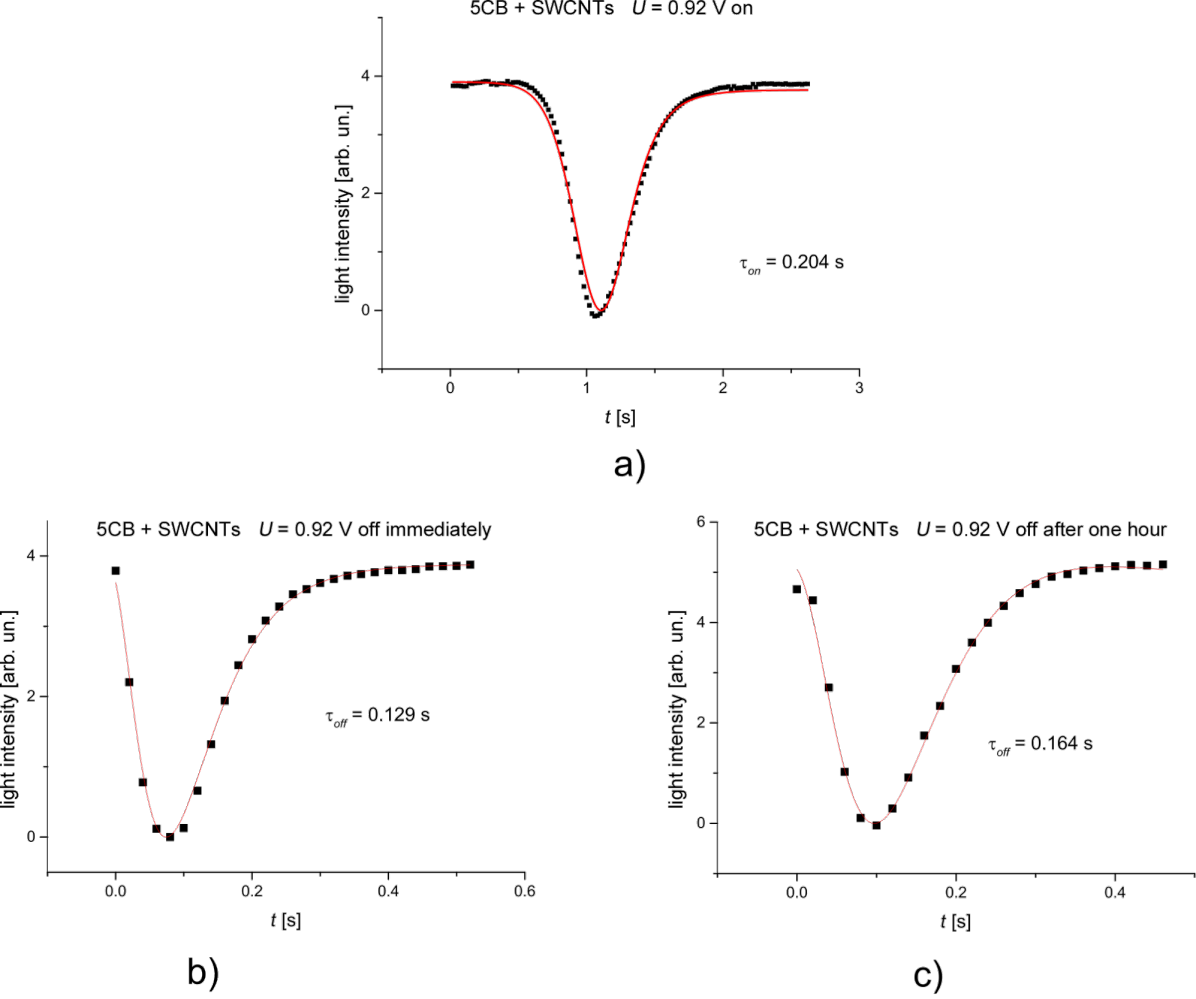
Variation of the intensity of a light beam traversing a cell containing LC + SWCNTs. a) The field is switched on, b) the field is switched off immediately, c) the field is switched off after one hour.

Since the theoretical evaluation considered only small deviation angles, the first intensity maxima from Figure 6 were chosen for both samples. They are at 0.91 V for LC and 0.92 V for LC + SWCNTs. An acquisition system was used to record the emergent signal at every 20 ms. As it can be seen from Figure 7 and Figure 8 there is a good agreement between experimental data and the theoretical fitting function confirming our assumption for the LC cell and the LC + SWCNTs cell.

The calculated relaxation times when the field is switched on or off provided from the fitting parameters are similar to the times obtained from experimental data. Table 1 shows the results for the pure nematic and Table 2 for LC + SWCNTs where (τ_off_)*_I_* is the relaxation time when the field is switched off immediately and (τ_off_)*_L_* is the relaxation time when the is switched off after one hour.

**Table 1:** Relaxation times of 5CB.

	τ_on_ [s]	τ_off_ [s]

theoretical	0.173	0.159
experimental	0.173	0.158

**Table 2:** Relaxation times of the 5CB + SWCNTs mixture.

	τ_on_ [s]	(τ_off_)*_I_* [s]	(τ_off_)*_L_* [s]

theoretical	0.194	0.138	0.181
experimental	0.204	0.129	0.164

When the field is switched on, the molecules begin to align with the field but the interaction forces with the nanotube surfaces pull them back. This might explain why the Fréedericksz transition threshold and the relaxation times of the SWCNT-containing samples are higher than those of the pure nematic. When the field is switched off immediately, the anchoring forces act in the same direction as the elastic molecular forces making the molecules move faster to their original position. Other dynamic measurements performed on similar systems [25–26] confirm this faster response. Other publications [27–30] show the influence of the addition of nanotubes on the dielectric properties of the sample. In [27,29], Basu and co-workers clearly explained that the increase of dielectric anisotropy is strongly related to the anchoring forces. When the field is applied for a longer period of time, the nanotubes are all aligned to the field, as confirmed by other studies [19,21,27]. When the field is switched off, the nanotubes turn back to their original position slower than nematic molecules and the anchoring forces on their surface hinder a complete molecular relaxation leading to longer response times. This interaction model is confirmed by other independent studies [31–32].

## Conclusion

We present a simplified theoretical model to explain the behavior of nematic liquid crystals with added carbon nanotubes in an external electric field. This model is in good agreement with our experimental results but also with other published studies. Thus, a proper characterization of these nanomaterial compounds can be made and a deeper understanding of the anchoring forces influence on the physical properties of the mixture is provided.
